# CARF promotes spermatogonial self-renewal and proliferation through Wnt signaling pathway

**DOI:** 10.1038/s41421-020-00212-7

**Published:** 2020-11-17

**Authors:** Wenhao Cui, Xiaoli He, Xiaohong Zhai, Huan Zhang, Yuanwei Zhang, Fei Jin, Xiaomin Song, Dianqing Wu, Qinghua Shi, Lin Li

**Affiliations:** 1 State Key Laboratory of Molecular Biology, CAS Center for Excellence in Molecular Cell Science, Shanghai Institute of Biochemistry and Cell Biology, Chinese Academy of Sciences, University of Chinese Academy of Sciences, Shanghai, 200031 China; 2 School of Life Science and Technology, Shanghai Tech University, Shanghai, 201210 China; 3 School of Life Sciences, University of Science and Technology of China, Hefei, Anhui 230027 China; 4 Vascular Biology and Therapeutic Program and Department of Pharmacology, Yale School of Medicine, New Haven, CT 06520-8089 USA; 5 School of Life Science, Hangzhou Institute for Advanced Study, University of Chinese Academy of Sciences, Hangzhou, Zhejiang 310024 China

**Keywords:** Stem-cell niche, Morphogen signalling, Self-renewal

## Abstract

Collaborator of ARF (CARF) regulates cell proliferative fate through both p53-dependent and -independent mechanisms. Recently, we reported a new function of CARF as a positive regulator of Wnt signaling. Despite these findings, the physiological function of CARF has not been well studied. Here, we generated *CARF* knockout mice and found that male *CARF*^*−/−*^ mice exhibited significantly impaired fertility and Sertoli-cell-only (SCO) syndrome phenotypes. Further studies revealed that loss of CARF in Sertoli cells led to decreased GDNF expression, which hindered spermatogonial stem cells (SSCs) self-renewal. Meanwhile, CARF loss in undifferentiated spermatogonia impaired their proliferation. These two mechanisms together led to SCO syndrome phenotypes, which could be functionally rescued by pharmacological or genetic reactivation of Wnt signaling. Finally, we identified CARF^S351F^ as a potential pathogenic mutation in an SCO patient. Overall, our findings reveal important roles of CARF in spermatogonial self-renewal and proliferation through the Wnt signaling pathway.

## Introduction

Reproduction is critical for passing genetic information between generations for all mammalian species. An adult male continuously produces millions of haploid spermatozoa daily^1^. The process of spermatogenesis is under complex regulation of various regulators including external factors and internal factors^2^. However, detailed regulatory mechanisms have not been well understood. Infertility affects about 10–15% of reproductive-aged couples on a global scale, with male factors being the cause in approximately half^3^. Sertoli-cell-only (SCO) syndrome is one important cause of male infertility, in which only Sertoli cells line the seminiferous tubules without any germ cells. Although there is no accurate data at present, it is estimated that SCO patients may account for approximately 10% of infertile men. It is believed that SCO syndrome is caused by multiple factors, such as Y-chromosome micro-deletions, exposure to chemicals and toxins^4^. However, the etiology and detailed mechanisms underlying most cases of SCO syndrome remain largely unclear^5^.

Spermatogonial stem cells (SSCs) are responsible for giving rise to all stages of spermatids, although they account for 0.03% only of all germ cells in mice^2^. In order to maintain sperm supply for a long time, SSCs must balance self-renewal and proliferation, which is strictly regulated by extrinsic and intrinsic signaling factors. Abnormalities in these processes would lead to spermatogenic impairment and male infertility^6^. External signals mainly come from niches, where Sertoli cells are considered as a key cell population. Sertoli cells not only make up the blood-testis barrier but also secrete some growth factors, such as GDNF, SCF, FGF2 and BMP4, which are important for spermatogenesis^7^. Therefore, in addition to the internal regulation of gene expression in SSCs, the communication between SSCs and Sertoli cells is also crucial for SSC fate decisions.

CARF was initially identified as a collaborator of ARF^8^. Previous study showed that attenuated CARF expression could trigger DNA damage response and resulted in cell mitotic arrest and apoptosis; while overexpression of CARF could impair proliferation and contributes to senescence by activation of p53-HDM2-p21 pathway^9^. However, the superexpression of CARF would trigger pro-proliferation through interaction with ERK^10^. In addition, CARF is also suggested to function in cellular reprogramming and pre-ribosomal RNA processing^11,12^. Recently, we and another lab revealed a new role of CARF as a positive regulator in the Wnt signaling pathway^13,14^. However, these findings above are mostly based on studies at the cellular level or using mouse xenograft models, and the physiological function of CARF in vivo remains unknown.

In this study, we generated *CAR*F-knockout (KO) mice and revealed an important role of CARF in spermatogenesis. We found that *CARF* depletion attenuated Wnt signaling activity in Sertoli cells and undifferentiated spermatogonia, which then impaired SSCs self-renewal and undifferentiated spermatogonia proliferation, respectively. The combined effects led to germ cell loss and consequently a significantly decreased fertility in *CARF*^*−/−*^ mice.

## Results

### Inactivation of CARF leads to SCO syndrome

**Fig. 1:**
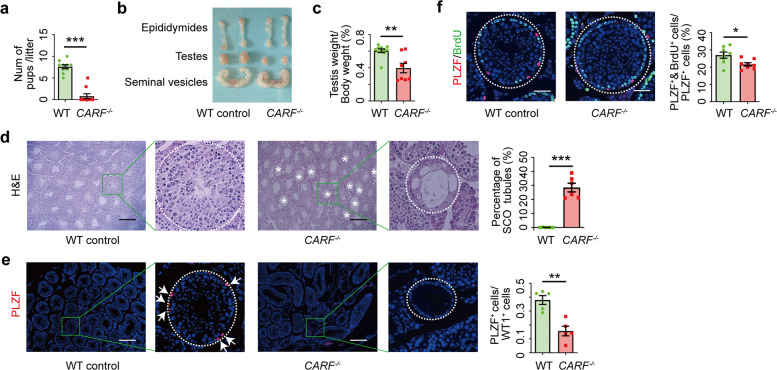
The depletion of *CARF* in male mice leads to SCO syndrome and impaired fertility. **a** Comparison of male fertility of WT control and *CARF*^*−/−*^ mice (*n* = 5) at 8–16 weeks of age. Each dot in the graphs represents an individual litter. Bar graphs represent means ± SEM. Statistical analysis was performed by two-tailed *t*-test, ***P < 0.001. **b** Gross morphology of representative testes, epididymides and seminal vesicles from 16-week-old WT control (left) and *CARF*^*−/−*^ mice (right). **c** Average testes weight of WT control mice (*n* = 11) and *CARF*^*−/−*^ (*n* = 8) at 8–16 weeks of age. Each dot in the graphs represents an individual mouse. Bar graphs represent means ± SEM. Statistical analysis was performed by two-tailed *t-*test, **P < 0.01. **d** H&E staining of representative testis sections from 16-week-old WT control and *CARF*^*−/−*^ mice (Scale bars, 200 μm). The boxed area is magnified on the right side. The white asterisk indicates an SCO seminiferous tubule. Dot graph shows the ratios of SCO seminiferous tubules in the testis sections from 8- to 16-week-old WT control and *CARF*^*−/−*^ mice (mice, *n* = 6; sections of one mouse, *n* > 5). Each dot in the graphs represents an individual mouse. Bar graphs represent means ± SEM. Statistical analysis was performed by two-tailed *t-*test, ***P < 0.001. **e** Immunostaining for undifferentiated spermatogonia (including SSCs) marker PLZF (red) in testis sections from 16-week-old WT control and *CARF*^*−/−*^ mice, with co-staining for DAPI (blue) (Scale bars, 200 μm). The boxed area is magnified on the right side. Dot graph shows the ratios between PZLF^+^ undifferentiated spermatogonia and Sertoli cells per seminiferous tubule in testis sections from 8- to 16-week-old WT control and *CARF*^*−/−*^ mice (mice, *n* = 5; tubules of one mouse, *n* ≥ 6). Each dot in the graphs represents an individual mouse. Bar graphs represent means ± SEM. Statistical analysis was performed by two-tailed *t*-test, **P < 0.01. **f** Cross-sectional images of PLZF (red) immunostaining, BrdU (green) immunostaining and DAPI (blue) staining of testis sections from 16-week-old WT control and *CARF*^*−/−*^ mice (Scale bars, 50 μm). Dot graph shows the percentage of BrdU^+^&PLZF^+^ cells in total PLZF^+^ cells of testis sections from 8- to 16-week-old WT control and *CARF*^*−/−*^ mice (mice, *n* = 8; tubules of one mouse, *n* ≥ 6). Each dot in the graphs represents an individual mouse. Bar graphs represent means ± SEM. Statistical analysis was performed by two-tailed *t-*test, *P < 0.05.

To explore the physiological function of CARF, we first examined the expression of CARF in multiple organs of mice by quantitative real-time PCR and western blot analysis. We found that CARF was highly expressed in the testis, thymus, and spleen (Supplementary, Fig. S1a, b). This result is consistent with the information from the UniGene database. Next, we generated *CARF*-KO mice using CRISPR/Cas9-mediated genome editing techniques (Supplementary, Fig. S1c), which was confirmed by DNA Sanger sequencing and western blot analysis (Supplementary, Fig. S1d, e). To prevent off-target effects, we crossed *CARF*^*+/−*^ male mice with the C57 female mice for 6 generations, and then the gene-edited mice were used in our subsequent studies. The proportion of three genotypes, wild-type (WT), CARF^*+/−*^ and *CARF*^*−/−*^ neonates, was in accordance with Mendel’s laws of inheritance in heterozygous mice breeding (results not shown), suggesting that inactivation of CARF does not cause embryonic lethality. While the *CARF*^*−/−*^ females had normal fertility, *CARF*^*−/−*^ males displayed incomplete penetrance of sterility, despite of their normal copulating behavior. Five 8-week-old *CARF*^*−/−*^ male mice and five WT littermate male mice were used for fertility testing by housing one male with two WT females. All five WT males gave birth (7.60 ± 0.45 pups/litter, *n* = 10; Fig. 1a). In contrast, only two *CARF*^*−/−*^ males sired one litter each after 21 days of detection of the plugs (3 and 5 pups/litter), and the remaining three *CARF*^*−/−*^ males did not produce any offspring. The testis-to-body weight ratios of male *CARF*^*−/−*^ mice was much lower than those of WT littermates (Fig. 1b, c). The numbers of sperms from caudal epididymis of *CARF*^*−/−*^ mice were significantly lower than those of the WT littermates (Supplementary, Fig. S1f, g). The Hematoxylin and Eosin (H&E) staining showed that testes from 16-week-old *CARF*^*−/−*^ mice exhibited an incompletely penetrant phenotype of abnormal seminiferous tubules, with a high proportion (26.33%) of SCO tubules (Fig. 1d), whereas other tubules appeared to contain spermatogenic cells from all stages of spermatogenesis. To further characterize the seminiferous tubule phenotypes, we employed immunostaining with antibodies to the germ cell marker DEAD-box helicase 4 (MVH) and Wilms tumor protein (WT1), a marker for Sertoli cells. Consistent with the histological study, the staining showed that some of the seminiferous tubules in *CARF*^*−/−*^ testes were completely devoid of germ cells, with only Sertoli cells left (Supplementary, Fig. S1h, i). Next, we investigated whether there were germ cell phenotypes in those non-SCO seminiferous tubules. Immunostaining for undifferentiated spermatogonia (including SSCs) marker promyelocytic leukemia zinc-finger (PLZF) in testis, we found that the number of PLZF-positive undifferentiated spermatogonia was significantly reduced in the non-SCO tubules of *CARF*^*−/−*^ testes compared with that in the WT controls (Fig. 1e). Moreover, we administered a short-duration (2 h) 5-Bromo-2′-deoxyuridine (BrdU) pulse to WT and *CARF*^*−/−*^ mutant males, and found a markedly lower ratio of BrdU- and PLZF-double positive cells to PLZF-positive cells (BrdU^+^&PLZF^+^/PLZF^+^) in *CARF*^*−/−*^ testes compared with WT controls (Fig. 1f). These observations indicate a proliferative defect for the undifferentiated spermatogonia in the non-SCO seminiferous tubules of *CARF*^*−/−*^ testes. Of note, we did not observe specific arrests of spermatogenesis or increase of cell apoptosis in *CARF*^*−/−*^ testes (Supplementary, Fig. S1j), indicating that there are no significant defects in the later stages of spermatogenesis. Overall, these studies revealed that the ablation of *CARF* results in SCO syndrome phenotypes and causes impaired spermatogenesis, indicating an important role of CARF in spermatogenesis.

### *CARF*^−/−^ mice develop age-dependent seminiferous tubule structural defect

**Fig. 2:**
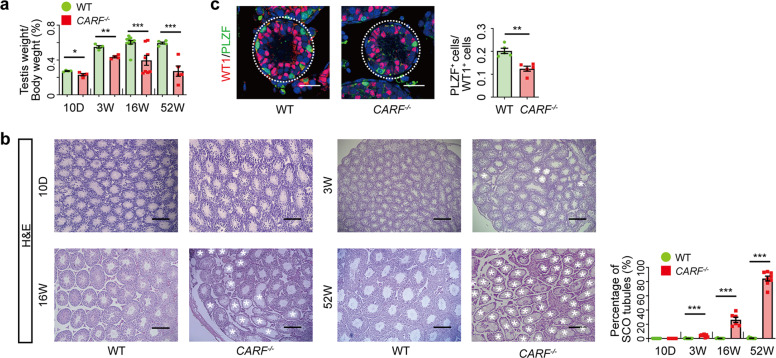
*CARF*^*−/−*^ mice develop age-dependent seminiferous tubule damage. **a** Average testes weight of 10-day-old, 3-week-old, 16-week-old, 52-week-old WT control or *CARF*^*−/−*^ mice (10-day-old mice, *n* = 4; 3-week-old mice, *n* = 4; 16-week-old mice, *n* ≥ 8; 52-week-old, *n* = 5). Each dot in the graphs represents an individual mouse. Bar graphs represent means ± SEM. Statistical analysis was performed by two-tailed *t*-test, *P < 0.05, **P < 0.01,***P < 0.001. (**b**) H&E staining of representative testis sections from 10-day-old, 3-week-old, 16-week-old, 52-week-old WT control and *CARF*^*−/−*^ mice (Scale bars for 10D, 100 μm, for others, 200 μm). The white asterisk indicates an SCO seminiferous tubule. Dot graph shows the percentage of SCO tubules in testis sections from 10-day-old, 3-week-old, 16-week-old, 52-week-old WT control and *CARF*^*−/−*^ mice (10-day-old mice, *n* = 8; 3-week-old mice, *n* = 8; 16-week-old mice, *n* = 6; 52-week-old, *n* = 8, sections of one mouse, *n* > 5). Each dot in the graphs represents an individual mouse. Bar graphs represent means ± SEM. Statistical analysis was performed by two-tailed *t-*test, ***P < 0.001. **c** Immunostaining for undifferentiated spermatogonia marker PLZF (green) and Sertoli cell marker WT1 (red) in testis sections from 10-day-old WT control and *CARF*^*−/−*^ mice, with co-staining for DAPI (blue) (Scale bars, 20 μm). Dot graph shows the ratios between PZLF^+^ undifferentiated spermatogonia and Sertoli cells per seminiferous tubule in testis sections from 10-day-old WT control and *CARF*^*−/−*^ mice (mice, *n* = 5; tubules of one mouse, *n* ≥ 6). Each dot in the graphs represents an individual mouse. Bar graphs represent means ± SEM. Statistical analysis was performed by two-tailed *t*-test, **P < 0.01.

*CARF*^*−/−*^ male mice exhibited subfertility at 8–16 weeks of age, and when they aged to 52–week, they were completely infertile. To explore the process of aging-related fertility decline, we examined mice at different ages. The testicular weights had shown a difference between *CARF*^*−/−*^ mice and their WT littermates at 10 days, and the difference became greater with age (Fig. 2a). H&E staining results indicated that SCO syndrome phenotypes could not be observed from the 10-day-old mice testis sections, but were detected at 3-week-old mice testis sections. Almost all the seminiferous tubules in *CARF*^*−/−*^ mice had become SCO at 52 weeks of age; in contrast, the all WT control littermates had normal seminiferous tubules that contained all stages of germ cells (Fig. 2b). Although SCO syndrome phenotypes were not observed in 10-day-old male mice testis sections, there was a difference in the number of undifferentiated spermatogonia. Testis cross-sectional images of PLZF and WT1 immunostaining showed that PLZF^+^ undifferentiated spermatogonia were reduced in *CARF*^−/−^ mice compared with WT controls (Fig. 2c). These observations suggest that the reproductive defects in *CARF*^*−/−*^ mice progress as they get older. It also implies that the cause of the defect play a role in infancy and continue to function in adulthood.

### Decreased GDNF expression in *CARF*-null Sertoli cell causes defective SSC self-renewal

We next set out to investigate possible mechanisms underlying the SCO phenotypes observed in the *CARF*-null mice. Since the hypothalamic-pituitary-gonadal axis (HPG) plays a critical role in spermatogenesis^15^, we first investigated whether the spermatogenic phenotype of *CARF* KO mice is due to disruption of the endocrine axis. For this, we evaluated the effects of *CARF* depletion on pituitary, Leydig cell development and seminal vesicles, in all of which we did not observe any apparent phenotypes (data were not shown). Since Follicle-stimulating hormone (FSH) and testosterone are the key gonadotrophins, we then analyzed serum FSH and testosterone levels in WT and *CARF*^*−/−*^ mice. As shown in Supplementary, Fig. S2a, b, we did not observe significant differences in serum FSH and testosterone levels between WT and *CARF*^*−/−*^ mice at puberty. Of note, at puberty, SCO phenotype in *CARF*^*−/−*^ mice has already been clearly manifested. These findings suggest that spermatogenesis defects in *CARF*^*−/−*^ mice do not mainly attribute to hormonal imbalances.

**Fig. 3:**
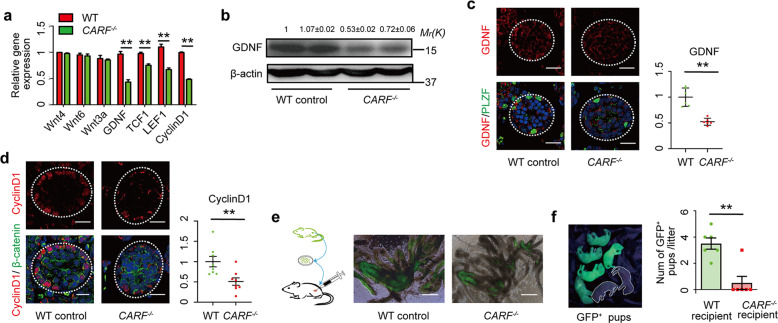
Decreased GDNF expression in Sertoli cells causes defective SSC self-renewal. **a** Quantitative real-time PCR analysis of mRNA levels of *Wnts* and Wnt target genes in Sertoli cells from 10-day-old WT control and *CARF*^*−/−*^ mice. Data are presented as means ± SEM. Fold changes were compared with WT controls, and are normalized to β-actin. **b** Western blot analysis of GDNF in Sertoli cells from WT control and *CARF*^*−/−*^ mice. Quantification of blotting intensity for indicated proteins is shown on the top (The first WT sample is set as 1.0 after normalization with β-actin blotting). **c** Immunostaining for GDNF (red) in testis sections from 10-day-old WT control and *CARF*^*−/−*^ mice, with co-staining for undifferentiated spermatogonial marker PLZF (green) and DAPI (blue) (Scale bars, 20 μm). The outlines of seminiferous tubules are indicated by dashed lines. Dot graph shows the relative intensities of immunostaining for GDNF, data are presented as means ± SEM. Statistical analysis was performed by two-tailed *t-*test, **P < 0.01. **d** Testis cross-sectional images of β-catenin (green) immunostaining, CyclinD1 (red) immunostaining and DAPI (blue) staining of WT control and *CARF*^*−/−*^ mice at 10 days of age (Scale bars, 20 μm). The outlines of seminiferous tubules are indicated by dashed lines. Dot graph shows the relative intensities of immunostaining for CyclinD1, data are presented as means ± SEM. Statistical analysis was performed by two-tailed *t*-test, **P < 0.01. **e** The donor-derived GFP-positive colonies within seminiferous tubule of recipient testis 2 months after transplantation. **f** GFP-positive pups were produced by donor cell-derived sperm. Comparison of the number of GFP-positive pups of WT and *CARF*^*−/−*^ recipient (*n* = 3) that was transplanted with WT GFP-positive germ cells and mated with a WT female mouse within 2 months. Each dot in the graphs represents an individual litter. Bar graphs represent means ± SEM. Statistical analysis was performed by two-tailed *t-*test, **P < 0.01.

Previously, we and another group have reported the role of CARF in canonical Wnt signaling^13,14^. It is known that Wnt signaling directly regulates GDNF expression in Sertoli cells^16^. GDNF is an important factor secreted by Sertoli cells, which controls SSC fate in a dose-dependent manner. Inhibition of GDNF signaling by pharmacological or genetic methods will lead to depletion of undifferentiated SSCs and subsequently reduced mature spermatogonia, which results in SCO seminiferous tubules^2,6,17,18^. Therefore, we asked whether the depletion of *CARF* might lead to impaired fertility through the Wnt-GDNF axis. To investigate this possibility, we first checked the expression of CARF in Sertoli cells and spermatogonia of WT mice by immunofluorescence staining, which showed that CARF is expressed in Sertoli cells and undifferentiated spermatogonia (Supplementary, Fig. S2c, d). This result is consistent with what Tong’s lab has obtained by single-cell RNA sequencing, which also showed that CARF is expressed in undifferentiated spermatogonia^19^. Next, we examined Wnt activity and GDNF expression in *CARF*^*−/−*^ mice by quantitative real-time PCR. Sertoli cells also secrete other growth factors in addition to GDNF and these factors also play important roles in the maintenance of SSCs^7,20,21^. We thus included two other Sertoli cell-secreted hormones, Wnt4 and Wnt6, for investigation. As shown in Fig. 3a, expression of GDNF and Wnt target genes including *Tcf1* and *CyclinD1* were significantly reduced upon depletion of *CARF*; while *Wnt4* and *Wnt6* expression were not apparently affected (Fig. 3a). Protein analysis by western blot and immunofluorescent staining confirmed the alteration in protein expression levels detected by quantitative real-time PCR (Fig. 3b, c). Testis cross-sectional immunostaining for CyclinD1 revealed that Wnt signaling activity decreased in Sertoli cells of *CARF*^*−/−*^ male mice, while either β-catenin protein levels or its nuclear distribution did not change significantly (Fig. 3d). This is consistent with our previous findings that CARF does not affect β-catenin accumulation and distribution^14^. To further clarify the effect of GDNF decline on spermatogenesis and fertility, we transplanted GFP^+^ germ cells from WT testes into the busulfan-treated WT control and busulfan-treated *CARF*^*−/−*^ mice. Compared with WT recipient testis, *CARF*^*−/−*^ recipients have fewer donor-derived GFP-positive colonies within seminiferous tubule (Fig. 3e). In addition, crosses between recipient males and WT females indicated the number of GFP-positive pups produced by donor-derived germ cells from *CARF*^*−/−*^ recipients is significantly less than WT recipients (Fig. 3f). Together, these results suggest that *CARF* depletion reduces GDNF expression in Sertoli cells through the canonical Wnt signaling, which further leads to inhibition of SSCs self-renewal.

### Restoring CARF in Sertoli cells promotes GDNF expression and SSC self-renewal

**Fig. 4:**
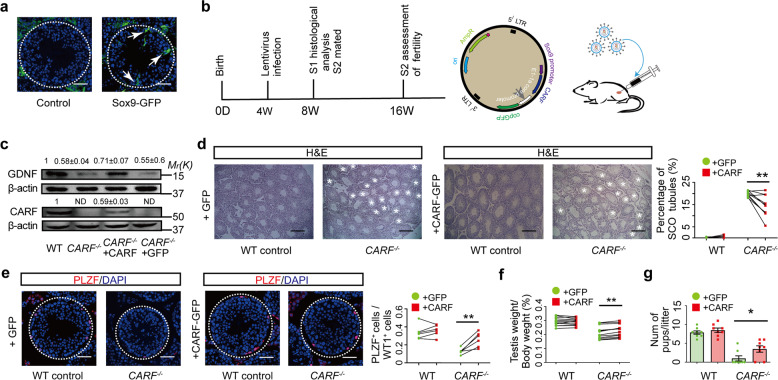
Restoring CARF in Sertoli cells promotes GDNF expression and SSC self-renewal. **a** The GFP expression in seminiferous tubule driven by the Sox9 promoter from freezing sections of testis were infected with lentivirus (Scale bars, 50 μm). The outlines of seminiferous tubules are indicated by dashed lines. Of note, Leydig cells have autofluorescence which is defined as variable, background fluorescence that can be detected in multiple channels. **b** Schematic strategy for rescuing CARF expression in Sertoli cells by lentivirus. **c** Western blot analysis for CARF and GDNF expression in the testes from 8-week-old WT control, *CARF*^*−/−*^ mice, and *CARF*^*−/−*^ mice were infected with lentivirus. Quantification of blotting intensity for indicated proteins is shown (The WT sample is set as 1.0 after normalization with β-actin blotting). **d** H&E staining of representative testis sections from 8-week-old WT and *CARF*^*−/−*^ mice after lentivirus infection (Scale bars, 200 μm). The white asterisk indicates an SCO seminiferous tubule. Dot graph shows the percentage of SCO seminiferous tubules from 8-week-old WT and *CARF*^*−/−*^ mice testis sectional after lentivirus infection (mice, *n* = 10; sections of one mouse, *n* > 5). Each dot in the graphs represents an individual testis from one mouse. Bar graphs represent means ± SEM. Statistical analysis was performed by paired *t*-test, **P < 0.01. **e** Testis sectional images of PLZF (red) immunostaining and DAPI (blue) staining of WT and *CARF*^*−/−*^ mice after lentivirus infection at 8 weeks of age (Scale bars, 50 μm). Dot graph shows the ratios between PZLF^+^ undifferentiated spermatogonia and Sertoli cells per seminiferous tubule in testis sections from lentivirus-infected WT and *CARF*^*−/−*^ mice. Each dot in the graphs represents an individual testis from one mouse. Bar graphs represent means ± SEM. Statistical analysis was performed by paired *t-*test, **P < 0.01. **f** The average weight of the testis from 8-week-old WT and *CARF*^*−/−*^ mice after lentivirus infection (*n* = 12). Each dot in the graphs represents an individual testis from one mouse. Bar graphs represent means ± SEM. Statistical analysis was performed by paired *t-*test, **P < 0.01. **g** Comparison of male fertility of WT and *CARF*^*−/−*^ mice after lentivirus infection (8–16 weeks, *n* = 8). Each dot in the graphs represents an individual litter. Bar graphs represent means ± SEM. Statistical analysis was performed by two-tailed *t-*test, *P < 0.05.

To further confirm the function of CARF in Sertoli cells in vivo, we designed a plasmid construct that expresses CARF under the control of the SOX9 promoter and then used this construct to restore CARF expression only in Sertoli cells. First, we confirmed that the SOX9 promoter is only active in Sertoli cells after testis infected by lentivirus (Fig. 4a). The plasmid also expressed copGFP driven by the Ef-1α promoter following the CARF coding sequence (Fig. 4b), allowing for measurement of lentiviral infection efficiency in the seminiferous tubules. Two months after the injection of the lentivirus expressing CARF into the testes, the GDNF expression in Sertoli cells was restored (Fig. 4c). Meanwhile, the SCO syndrome phenotypes (Fig. 4d) and the number of PLZF^+^ undifferentiated spermatogonia (including SSCs) around copGFP-positive Sertoli cells were improved (Fig. 4e). The size of testis and fertility were also improved (Fig. 4f, g). Together, these results support that CARF loss in Sertoli cells is responsible for the reduced GDNF expression and decreased SSC self-renewal in *CARF*^*−/−*^ mice.

### Ablation of *CARF* in undifferentiated spermatogonia results in reduced proliferation of undifferentiated spermatogonia

**Fig. 5:**
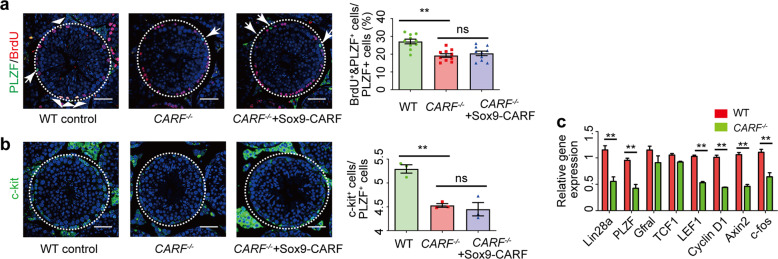
Ablation of *CARF* in undifferentiated spermatogonia results in reduced proliferation of undifferentiated spermatogonia. **a** Cross-sectional images of PLZF (green) immunostaining, BrdU (red) immunostaining and DAPI (blue) staining of WT control*, CARF*^*−/−*^ and *CARF*^*−/−*^ + SOX9-CARF mice (*CARF*^*−/−*^ mice was infected with lentivirus expressing CARF specifically in Sertoli cells) at 8 weeks of age (Scale bars, 50 μm). The outlines of seminiferous tubules are indicated by dashed lines. Arrowheads indicate PLZF^+^&BrdU^+^ cells, and arrows indicate PLZF^+^ BrdU^-^ cells. Dot graph shows the percentage of BrdU^+^&PLZF^+^ cells in total PLZF^+^ cells of testis sections from 8-week-old WT control*, CARF*^−*/−*^ and *CARF*^*−/−*^ + SOX9-CARF mice (mice, *n* = 10; tubules of one mouse, *n* ≥ 6). Each dot in the graphs represents an individual mouse. Bar graphs represent means ± SEM. Statistical analysis was performed by two-tailed *t*-test, n.s., not significant, **P < 0.01. **b** Cross-sectional images of differentiating spermatogonial cells marker c-Kit (green) immunostaining and DAPI (blue) staining of testis sections from 8-week-old WT control, *CARF*^*−/−*^ and *CARF*^*−/−*^ + SOX9-CARF mice (Scale bars, 50 μm). Dot graph shows the ratios between c-Kit^+^ cells and PLZF^+^ cells per seminiferous tubule in testis sections from 8-week-old WT control, *CARF*^*−/−*^ and *CARF*^*−/−*^ + SOX9-CARF mice (mice, *n* = 3; tubules of one mouse, *n* ≥ 10). Each dot in the graphs represents an individual mouse. Bar graphs represent means ± SEM. Statistical analysis was performed by two-tailed *t-*test, n.s., not significant, **P < 0.01. **c** Quantitative real-time PCR analysis of mRNA levels of SSC markers in testis and Wnt target genes in SSCs from WT control and *CARF*^*−/−*^ mice. Data are expressed as means ± SEM. Fold changes were compared with WT control, and normalized to Rps2.

A previous report showed that GDNF blocks SSC differentiation to maintain SSC pool, while it does not affect the proliferation of SSC^18^. Another study indicated that Wnt/β-catenin signaling in Axin2-positive undifferentiated spermatogonia regulates undifferentiated spermatogonial proliferation in vivo^22^. Therefore, we asked whether CARF loss-induced Wnt signaling alteration in undifferentiated spermatogonia might also contribute to the impaired fertility in *CARF*^*−/−*^ mice. For this, we first re-expressed CARF in Sertoli cells and tested whether it can rescue undifferentiated spermatogonia proliferation defect in *CARF*-null mice. We found that the number of PLZF^+^ undifferentiated spermatogonia of *CARF*^*−/−*^ mice could be rescued; however, the proliferation of undifferentiated spermatogonia could not be rescued by restoring CARF expression in Sertoli cells as demonstrated by the BrdU incorporation assay (Fig. 5a). Consistent with this, there were fewer c-Kit^+^ (differentiating spermatogonial cell marker) cells in seminiferous tubules per tubule in *CARF*^*−/−*^ mice than in WT control, which could not be rescued by restoring CARF expression in Sertoli cells either (Fig. 5b). These results suggest that the depletion of *CARF* in Sertoli cells is not the only mechanism underlying the reduced undifferentiated spermatogonia proliferation. To test whether *CARF* depletion may affect undifferentiated spermatogonia proliferation directly through down-regulating Wnt signaling in undifferentiated spermatogonia, we examined Wnt signaling activity in undifferentiated spermatogonia of *CARF*^*−/−*^ mice. Quantitative real-time PCR analysis demonstrated that the Wnt signaling activity in undifferentiated spermatogonia was indeed attenuated, and the undifferentiated spermatogonial markers PLZF and Lin28a in testis were also reduced (Fig. 5c). These data imply that the deletion of CARF causes defective proliferation of undifferentiated spermatogonia by directly down-regulating Wnt signaling autonomously.

### Restoration of Wnt signaling substantially rescues fertility defects in *CARF*^−/−^ mice

**Fig. 6:**
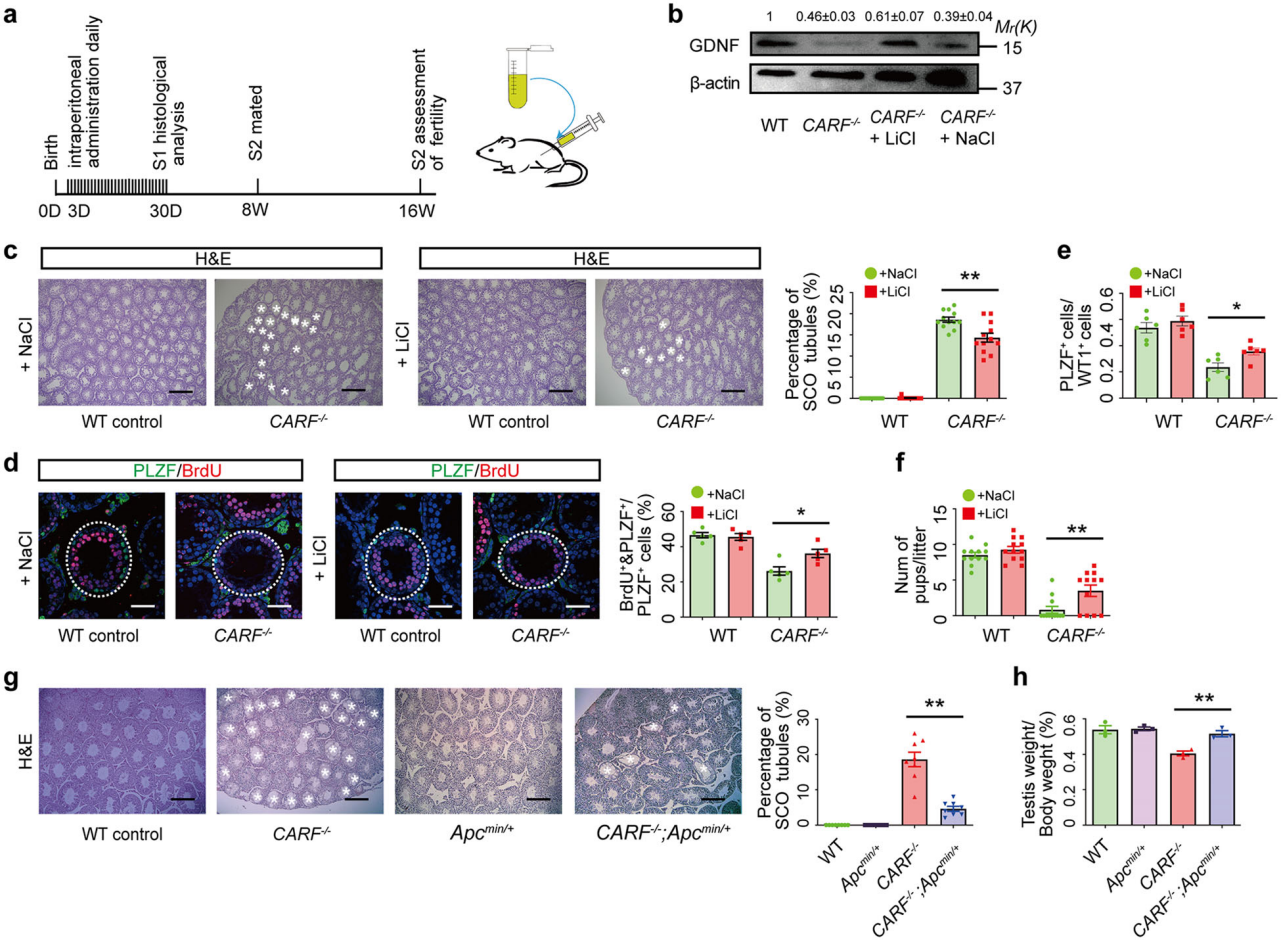
Restoration of Wnt signaling substantially rescues fertility defects in *CARF*^−/−^ mice. **a** Schematic illustration of the drug administration scheme. **b** Total testis lysates from *CARF*^−*/−*^ mice after drug treatment for 4 weeks were analyzed for GDNF expression by western blotting, with β-actin as a loading control. Quantification of blotting intensity for indicated proteins is shown (The WT sample is set as 1.0 after normalization with β-actin blotting). **c** H&E staining of representative testis sections from 4-week-old WT control and *CARF*^−/−^ mice after drug treatment (Scale bars, 200 μm). The white asterisk indicates an SCO seminiferous tubule. Dot graph shows the percentage of SCO seminiferous tubules in testis sections from 4-week-old WT control and *CARF*^*−/−*^ mice (mice, *n* = 12; sections of one mouse, *n* > 5) after drug treatment. Each dot in the graphs represents an individual mouse. Bar graphs represent means ± SEM. Statistical analysis was performed by two-tailed *t-*test, **P < 0.01. **d** Cross-sectional images of PLZF (green) immunostaining, BrdU (red) immunostaining and DAPI (blue) staining of testis sections from 4-week-old WT control and *CARF*^−/−^ mice after drug treatment (Scale bars, 50 μm). The outlines of seminiferous tubules are indicated by dashed lines. Dot graph shows the percentage of BrdU^+^&PLZF^+^ cells in total PLZF^+^ cells of testis sections from 4-week-old WT control and *CARF*^*−/−*^ mice after drug treatment (mice, *n* = 5; tubules of one mouse, *n* ≥ 6). Each dot in the graphs represents an individual mouse. Bar graphs represent means ± SEM. Statistical analysis was performed by two-tailed *t*-test, *P < 0.05. **e** Ratios between PZLF^+^ undifferentiated spermatogonia and Sertoli cells per seminiferous tubule in testis sections from 4-week-old WT control and *CARF*^−*/−*^ mice after drug treatment (mice, *n* = 5; tubules of one mouse, *n* ≥ 6). Each dot in the graphs represents an individual mouse. Bar graphs represent means ± SEM. **f** Comparison of male fertility in WT control and *CARF*^*−/−*^ mice after drug treatment (8–16 weeks, *n* = 6). Each dot in the graphs represents an individual litter. Bar graphs represent means ± SEM. Statistical analysis was performed by two-tailed *t-*test, **P < 0.01. **g** H&E staining of representative testis sections from 16-week-old WT*, CARF*^−*/−*^, *APC*^*min/+*^ and *CARF*^*−/−*^; *APC*^*min/+*^ mice (Scale bars, 200 μm). Percentage of SCO seminiferous tubules from 8- to 16-week-old WT, *CARF*^*−/−*^, *APC*^*min/+*^ and *CARF*^*−/−*^; *APC*^*min/+*^ testis sectional (mice, *n* = 8; sections of one mouse, *n* > 5). Each dot in the graphs represents an individual mouse. Bar graphs represent means ± SEM. Statistical analysis was performed by two-tailed *t*-test, **P < 0.01. **h** The average weight of testes from 8- to 16-week-old WT, *CARF*^*−/−*^, *APC*^*min/+*^, and *CARF*^*−/−*^; *APC*^*min/+*^ mice (*n* = 3). Each dot in the graphs represents an individual mouse. Bar graphs represent means ± SEM. Statistical analysis was performed by two-tailed *t-*test, **P < 0.01.

According to our findings above, ablation of *CARF* causes SCO syndrome through down-regulating Wnt signaling in two cell types: in Sertoli cells, down-regulation of Wnt signaling by depletion of *CARF* leads to a decrease of GDNF expression and subsequent impairment of SSC self-renewal; while in undifferentiated spermatogonia, it leads to defective cell proliferation of undifferentiated spermatogonia. To ask whether the SCO syndrome phenotype could be restored by reactivation of Wnt/β-catenin signaling, we used both chemical method with lithium chloride (LiCl) and genetic approach to boost Wnt signaling in *CARF*^*−/−*^ mice. LiCl is an activator of the canonical Wnt signaling pathway by inhibiting GSK3 activity. We treated *CARF*^*−/−*^ mice with LiCl (10 mg/kg body weight) daily from the third day of birth with NaCl as a negative control (Fig. 6a). We found that the expression of GDNF was increased after 4 weeks of LiCl treatment (Fig. 6b). Meanwhile, the SCO symptom was significantly improved, and the proportion of SCO seminiferous tubules decreased dramatically after the administration of LiCl (Fig. 6c). Consistent with this, the number of PLZF^+^ undifferentiated spermatogonia (including SSCs) and proliferation of undifferentiated spermatogonia were partly recovered (Fig. 6d, e). Meanwhile, the fertility defects in *CARF*^*−/−*^ male mice were also improved (Fig. 6f). Next, we used a genetic approach to activate Wnt signaling by crossing *CARF*^*−/−*^ mice with *Apc*^*min/+*^ mice. The *Apc*^*min/+*^ mouse model is a spontaneous intestinal adenoma model with Wnt signaling continuously being activated in the whole body^23^. Our results showed that SCO defects and reduced testicular size caused by *CARF* inactivation could be improved by over-activation of Wnt signaling through the introduction of the *Apc* mutation (Fig. 6g, h). Together, these data demonstrate that CARF-mediated Wnt signaling plays an important role in spermatogonial self-renewal and proliferation.

As mentioned before, CARF was reported to be involved in the regulation of the p53-HDM2-p21 axis^9^. To examine whether the fertility defects in *CARF*^*−/−*^ may be related to ARF or p53, we crossed *CARF*^*−/−*^ mice with *ARF*^*−/−*^ or *p53*^*−/−*^ mice to generate *CARF*^*−/−*^; *ARF*^*−/−*^ and *CARF*^*−/−*^; *p53*^*−/−*^ double knockout (DKO) mice, respectively. We found that there was no SCO syndrome phenotypes observed in testis cross-sections of *ARF*^*−/−*^ and *p53*^*−/−*^ mice (Supplementary, Fig. S3a). In addition, further inactivation of ARF or p53 had no apparent effect on the distribution of SCO seminiferous tubules in *CARF*^*−/−*^; *ARF*^*−/−*^ or *CARF*^*−/−*^; *p53*^*−/−*^ DKO mice compared with that in *CARF*^*−/−*^ mice (Supplementary, Fig. S3a, b). Moreover, the fertility of *CARF*^*−/−*^; *ARF*^*−/−*^ DKO male mice remained significantly lower than that of *ARF*^*−/−*^ males. Similar results were observed in *CARF*^*−/−*^; *p53*^*−/−*^ DKO mice (Supplementary, Fig. S3c). Together, these results suggest that the p53/ARF pathway is not the main mechanism, if any, for the SCO phenotypes observed in *CARF*^*−/−*^ mice.

### Identification of a CARFS351F mutation in SCO syndrome patients

**Fig. 7:**
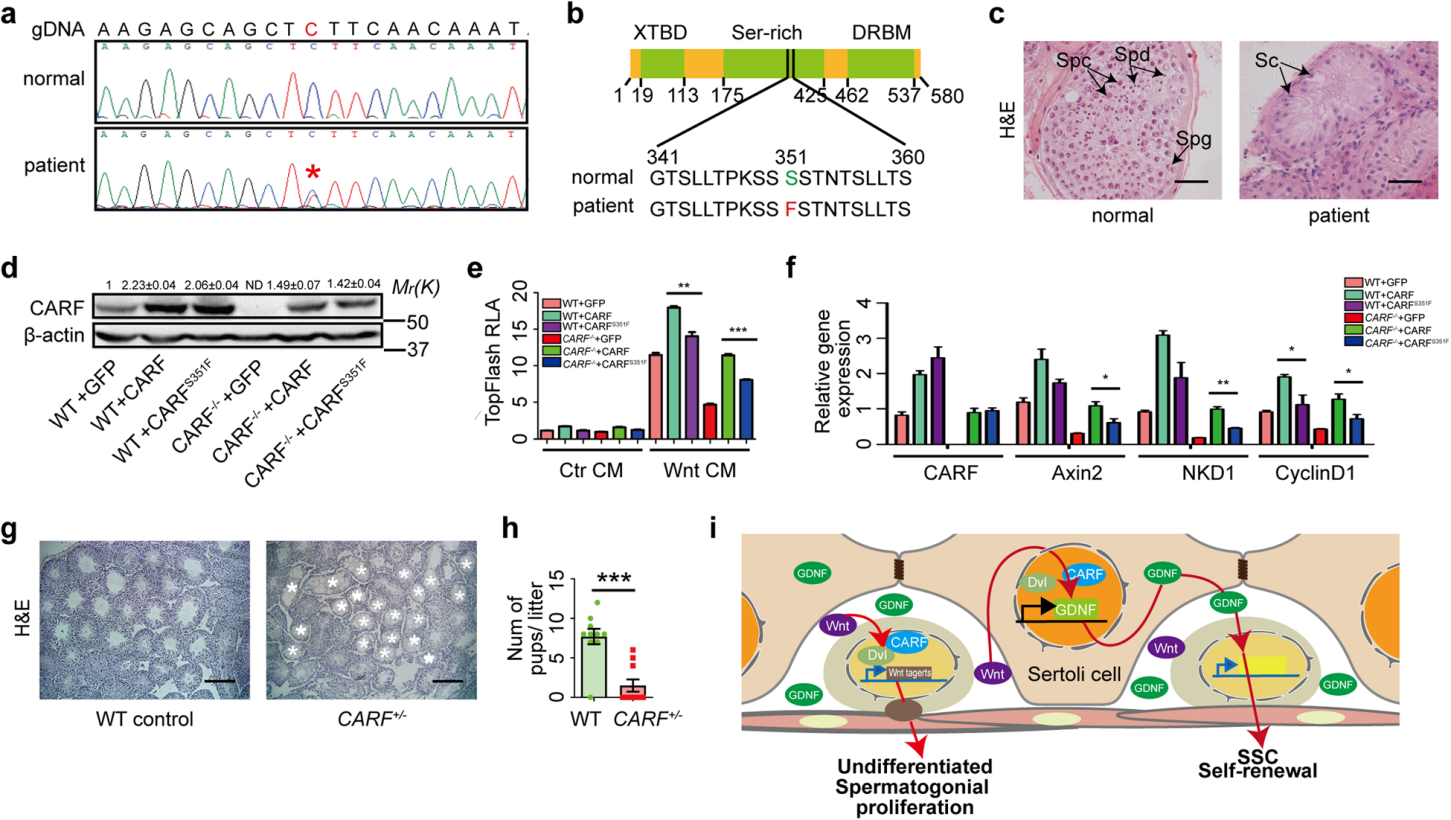
Identification of a CARF^S351F^mutation in SCO patients. **a** Sanger sequencing confirmed the CARF g.184367889 C > T mutation in the patient P2623 at the genomic DNA (gDNA) level. **b** Schematic representation of the domain structure of human CARF (top) and the altered amino acid identified in the mutant was colored (bottom). **c** H&E staining of testicular biopsies from patient P2623 and an obstructive azoospermia control. Spg, spermatogonia; Spc, spermatocyte; Spd, spermatid; Sc, Sertoli cell. (Scale bars, 15 μm). **d** Western blotting assays of CARF in WT and *CARF*^*−/−*^ U2OS cells after lentivirus infection, β-actin as a loading control. Quantification of blotting intensity for indicated proteins is shown (The WT sample is set as 1.0 after normalization with β-actin). **e** Effects of WT CARF and CARF^S351F^ on TOPflash reporter activity. Bar graphs represent means ± SEM. Statistical analysis was performed by two-tailed *t-*test, **P < 0.01. **f** Quantitative real-time PCR analysis of mRNA levels of Wnt target genes in WT and *CARF*^*−/−*^ U2OS cells after lentivirus infected. Data are expressed as means ± SEM. Fold changes were compared with WT U2OS cells infected by GFP-expressing lentivirus, and were normalized to GAPDH. **g** H&E staining of representative testis sections from 12-month-old WT control and *CARF*^*+/−*^ male mice (Scale bars, 200 μm). **h** Comparison of male fertility of 12-month-old WT control and *CARF*^*+/*−^ mice (*n* = 10). Each dot in the graphs represents an individual litter. Bar graphs represent means ± SEM. Statistical analysis was performed by two-tailed *t-*test, ***P < 0.001. **i** Working model showing how CARF regulates spermatogonial self-renewal and proliferation through Wnt signaling pathway.

To assess the potential significance of CARF function in human fertility, we screened for CARF mutations in an in-house whole exome sequencing (WES) data for 102 patients with SCO syndrome. A heterozygous missense mutation in patient P2623 was identified. There is no other mutation known to cause SCO syndrome in this patient (Supplementary, Fig. S4a). This mutation was subsequently verified by Sanger sequencing (Fig. 7a). The C to T transition leads to the conversion of Serine (S) to Phenylalanine (F) at Residue 351 (Fig. 7b). The testes of patient P2623 only had Sertoli cells without any visible germ cells; while healthy control testes contained different types of germ cells (Fig. 7c). The fertile controls had fathered at least one child. The patient underwent semen analyses, and excluded a history of orchitis, obstruction of vas deference, or other disorders (Supplementary, Fig. S4b).

To examine whether this S351F mutation may affect Wnt signaling, we constructed a plasmid vector expressing CARF^S351F^ and transfected it into WT and *CARF*^*−/−*^ U2OS cells (Fig. 7d). *CARF*-KO decreased Wnt3a-induced TOPflash reporter activity, whereas the CARF overexpression enhanced reporter activity. However, the expression of CARF^S351F^ was less effective in potentiating Wnt-induced activity than that of WT CARF (Fig. 7e). We next assessed the mRNA level of Wnt target genes by quantitative real-time PCR. Consistent with the results of the reporter assay, CARF^S351F^ was less effective in promoting Wnt-induced Axin2, NKD1, CyclinD1 expression than WT CARF (Fig. 7f). These results together suggest that S351F mutation compromises CARF’s ability to promoting Wnt signaling activity. Because the *CARF*^*+/−*^ mice showed markedly reduced fertility and increased proportion of SCO seminiferous tubules compared with WT littermates when they were at 12-month old (Fig. 7g, h), it is likely that the heterozygous CARF^S351F^ mutation may cause aging-related SCO syndrome.

## Discussion

CARF was initially identified as a partner of ARF to promote p53 activation. Further studies showed that CARF also functions through mechanisms independent of p53. Current knowledge about CARF function is mainly from studies based on cell lines or mouse xenograft models, and its physiological function in vivo remains unknown. Therefore, in this study, we generated *CARF*-KO mice and observed phenotypes of impaired fertility and SCO syndrome in *CARF*-null mice. We crossed *CARF*^*−/−*^ mice with *ARF*^*−/−*^ or *p53*^*−/−*^ mice; however, depletion of *ARF* or *p53* does not affect infertility and SCO syndrome phenotype of *CARF*^*−/−*^ mice. By contrast, activation of Wnt signaling by injection of LiCl or by crossing *CARF*-null mice with *APC*^*min/+*^ mice substantially rescues fertility defects in *CARF*^*−/−*^ mice. These findings suggest that CARF functions in spermatogonial self-renewal and proliferation mainly through the Wnt pathway rather than p53/ARF pathway.

The Wnt signaling pathway plays important roles in the male reproductive system. On the one hand, Wnt signaling exerts an antagonistic effect on testis-determining pathways in sex determination during the embryonic stage; on the other hand, it promotes sperm maturation in adult epididymis^24–27^. Many studies indicated that Wnt signaling is indispensable to SSCs function and fertility^28–30^, and plays a positive role in undifferentiated spermatogonia proliferation and maintenance^22,31^. According to our findings in this study, *CARF* depletion causes SCO syndrome in mice and improving Wnt signaling in *CARF*-null mice could to a large extent rescue SCO symptoms, suggesting that CARF-mediated Wnt signaling plays a positive role in both spermatogonial self-renewal and proliferation. Of note, our results showed that reactivation of Wnt signaling in *CARF*-null mice could only partially rescue infertility and SCO syndrome phenotypes. Thus, at the present stage, we cannot rule out the possibility that other mechanisms in addition to Wnt signaling may also contribute to these phenotypes observed in *CARF*-null mice.

In our study, we identified one heterozygous mutation CARF^S351F^ in SCO patients. Our study indicates that a mild loss-of-function of CARF, such as in the case of *CARF*^*+/−*^ heterozygous mice, is still able to induce SCO syndrome, but only does that after a long period of development. This indicates that time is also an important factor contributing to infertility. Therefore, patients harboring high-risk genotypes may reduce the adverse outcome of SCO syndrome by fathering at a young age. Considering the significance of Wnt signaling in male fertility, it is reasonable to expect mutations of Wnt pathway in SCO syndrome patients. However, except CARF^S351F^, we did not screen any other mutated genes in Wnt pathway from the examined patients. One explanation for this is probably due to the critical role of Wnt signaling in embryonic development, and mutations of the key players in Wnt signaling will normally lead to embryonic lethality. Our previous findings revealed CARF as a modulator of Wnt signaling rather than a key player like β-catenin^14^. Mutations towards modulators of Wnt signaling are supposed to have a less severe effects, and may cause observable defects after birth, such as in the case of *CARF*-KO. It is also possible that since CARF has a relatively high expression in testis, it may mediate a specific function of Wnt signaling in spermatogonial self-renewal and proliferation.

SCO syndrome, a medically refractory infertility disease, affects many couples in their child-bearing years. Although poly-genetic or environmental factors are reported to contribute to this defect, in most cases, specific causes and molecular mechanisms are not clear. There is no effective medical treatment for men with the diagnosis of SCO syndrome. These men may be able to reproduce under the condition of combined testicular sperm extraction with intracytoplasmic sperm injection. These are not offered as a standard treatment due to the low success rate: current reports show that only 13% of men receiving such procedures become a biological father^32^. There is considerable evidence suggesting that GDNF is essential for SSC self-renewal by blocking SSC differentiation. Singh et al reported that insufficient GDNF secretion may lead to infertility of some human patients with the phenotype of SCO^33^. We found that CARF loss could decrease GDNF expression by down-regulating Wnt signaling, thereby affecting spermatogenesis and fertility. Thus, our findings in this study provide a new strategy for diagnosing and treating infertility, and suggest that agonists of Wnt signaling might have potential clinical value for treating SCO syndrome.

## Materials and methods

### Antibodies

The following antibodies were used for immunostaining, or western blotting: mouse monoclonal anti-PLZF (Santa Cruz Biotech, Cat# sc-28319), rabbit polyclonal anti-PLZF (Santa Cruz Biotech, Cat# sc-22839), mouse monoclonal anti-γ-H2A (Millipore, Cat# 05-636), rabbit monoclonal anti-Cyclin D1 (Cell Signaling Technology, Cat# 2978), rat monoclonal anti-BrdU (Abcam Cat# ab6326), rabbit polyclonal anti-GDNF (Abcam, Cat# ab18956), rabbit polyclonal to anti-CDKN2AIP (Abcam, Cat# ab140519), rabbit polyclonal anti-MVH (Abcam, Cat# ab13840), rabbit monoclonal anti-Wilms Tumor Protein (Abcam, Cat# ab89901), mouse monoclonal anti-β-Catenin (BD Biosciences, Cat# 610154), goat Polyclonal anti-c-kit (R&D Systems, Cat# AF332-SP).

### Clinical sample information

SCO syndrome patients enrollment criteria for WES were set as (1) pathological examination showed that germ cells were absent with only Sertoli cells present in the seminiferous tubules of the testis; (2) no history of orchitis, pubertal parotitis, obstruction of vas deference, or endocrine disorders; (3) no AZF deletions in Y chromosome. The detailed clinical information of P2623 harboring CARF p.S351F mutation is listed in Supplementary, Fig. S4b.

### Mouse models and care

*CARF-*KO mouse model was generated using CRISPR-Cas9 technique by the Nanjing biomedical research institute of Nanjing University. For primers used for WT and *CARF*^*−/−*^ mice genotyping, see Supplementary, Table S1. The *p53*^*−/−*^ mice and the *ARF*^*−/−*^ mice were a generous gift from the Lijian Hui laboratory^34,35^. The *APC*^*min/+*^ mouse was obtained from the Nanjing biomedical research institute of Nanjing University^23^. Mice were housed in specific pathogen-free facilities. All animal experiment protocols and procedures were approved by the Shanghai Institute of Biochemistry and Cell Biology Animal Care and Use Committee and carried out under the guidelines.

### Quantitative real-time PCR assays

The total RNA was extracted with a TRIzol kit (Invitrogen, Cat# 15596026). Reverse transcription was performed using SuperScript III First-Strand Synthesis System (Thermofisher, Cat# 18080051) for RT-PCR according to the manufacturer’s instructions. Quantitative real-time PCR was performed with the SYBR Premix Ex Taq (Toyobo, Cat# QPK-201) on the QuantStudioTM 6 Flex real-time PCR system (Applied Biosystems, Waltham, MA, USA). Real-time PCR data were analyzed by the comparative CT method with use of β-actin or ribosomal protein S2 (Rps2) as the control for normalization. Primer sequences are listed in Supplementary, Table S1.

### Histological and immunofluorescence analyses

Testes and epididymides were fixed in Bouin’s solution (Sigma, Cat# HT10132-1L) overnight, and embedded in paraffin. Paraffin sections (3 μm) were stained with Hematoxylin and Eosin Staining Kit (Beyotime Biotechnology, Cat# C0105) following the instructions. Testes were fixed in 4% paraformaldehyde (PFA), embedded in paraffin and sectioned. To retrieve antigens, slides were boiled in Citrate Antigen Retrieval Solution for 15 min, and then incubated in a blocking buffer (5% Normal Donkey Serum) for 60 min at room temperature. The samples were subsequently stained with primary antibodies overnight at 4 °C and fluorescein-conjugated secondary antibodies for 2 h at room temperature. Nuclei were counterstained with DAPI mounting medium (Life Technologies). The images were captured with a BX51 fluorescence microscope (Olympus) or Fv1200 laser scanning microscope (Olympus).

### Isolation and culture of mouse Sertoli cells and SSCs

To isolate Sertoli cells, collagenase I (Worthington, Cat# LS004196) and trypsin were used for digestion of adult male seminiferous tubules that were cultured on lectin-coated plates^36^. Isolation of SSCs from the 5–7 days post-partum mice were performed by the protocols as Han’s lab described before^37^. Briefly, after Sertoli cells formed a confluent layer, they were treated with mitomycin C. The enriched SSCs were then cultured on the layer in MEMα basal medium supplemented with other components.

### Transplant experiments

Donor cells were obtained from 5–7 days old mice were derived by crossing B6.129SF1/J-Tg (ACTB-ECFP) CK6Nagy/J (gift from the Yi Arial Zeng laboratory). WT and *CARF*^*−/−*^ recipient mice were treated with busulfan (50 mg/kg) at 3 weeks of age to remove endogenous SSCs. After 6 weeks of busulfan treatment, donor cell suspensions from WT mice were injected into recipients with 15 μl volume (10^8^ cells/mL). GFP^+^ colonies into tubules were analyzed after 6 weeks and the fertility test was carried out after 8 weeks.

### Fertility experiment and Sperm mobility assays

For fertility experiment, pairs of one male mouse and two WT C57 female mice were caged together for mating purposes, female mice were randomly divided. Once the plug was detected, the female mouse was separated from the mating cage and raised separately. The number of pups per litter produced by each female was recorded.

For sperm mobility analysis, detailed operations were described before^38^. Briefly, the cauda epididymides were removed from surrounding adipose tissue, and then transferred to a dish containing 1 mL Enriched Krebs-Ringer bicarbonate medium pre-warmed to 37 °C. The sample was further chopped to release the sperm, which was adjusted to appropriate concentrations and dropped into the calibrated slide. Sperm number and mobility was finally assessed by using a computer-assisted semen analysis (CASA) machine (Leja).

### Serum hormone measurements

The blood was collected from the eyes around 2:00 PM from mice. Whole blood was added heparin and centrifuged for 15 min at 2500 × *g* to obtain plasma, then stored at −80 °C in case of hormonal analyses. The levels of serum testosterone were measured by competitive ELISA using commercial immunoassay kits (Testosterone mouse/rat ELISA Kit, LDN, Cat# AR E-8000). The levels of serum FSH were measured by sandwich ELISA using commercial immunoassay kits (FSH, Rodent, ELISA Kit, Abnova, Cat# KA2330). Absorbance at 450-630 nm was determined using an ELISA microtiter plate reader (BioTekInc., Winooski, VT, USA).

### Lentivirus packaging and testis transduction

For exogenous expression of CARF, we inserted the *CARF* complete coding sequence into the lentiviral vector of pCDH-CMV-MCS-EF1α-copGFP. Lentiviral packaging and concentrating were carried out by following standard procedures. In brief, the expression plasmid, the envelope plasmid pDM2G and the packaging plasmid psPAX2 were co-transfected to HEK-293T cells. Virus supernatant harvested after 48 h was centrifuged at 30,000 × *g* for 2 h and collected (titer of lentivirus > 10^8^ TU /mL). Lentivirus infection of mice testis was carried out as described before^39^. The 4-week-old male mice were anesthetized by intraperitoneal injections of pentobarbital-sodium. The subgroup1 (S1) mice for histological analysis, and the left and right testis in one mouse were infected with CARF or the same titer of GFP lentiviral respectively. After 4 weeks of injections, these mice were euthanized and testes were fixed in 4% PFA/PBS solution. The subgroup2 (S2) mice were randomly divided into two groups for fertility analysis. One group mice were infected with of CARF or the same titer GFP lentivirus, the left and right testis in the same mouse were infected with the same lentivirus. After 4 weeks of injected, these mice were mated with WT females of the C57 strain for the fertility tests.

### Drug administration

For activation of Wnt signal, LiCl (Sigma-Aldrich, St.Louis, MO) was dissolved in sterile water, and intraperitoneally injected to 3-day-old WT and *CARF*^*−/−*^ mice with a dosage of 10 mg/kg body weight. Littermate control *CARF*^*−/−*^ mice were injected with an equal amount of NaCl. The subgroup1 (S1) mice were anesthetized 30 days post-partum with carbon dioxide and then subjected to histological analysis. The subgroup2 (S2) mice were used for the fertility tests.

### BrdU incorporation and TUNEL assay

For BrdU labeling, BrdU was diluted with phosphate-buffered saline (PBS) to 10 mg/mL BrdU sterile solution. Mice were injected at a dose of 100 mg/kg. The mice were euthanized 2 h later and testes were fixed in 4% PFA/PBS solution for immunofluorescent staining.

Apoptotic cells were detected by In situ Cell Death Detection Kit (Roche, Cat# REF12156792910). All operations were performed according to the protocol. In short, after de-paraffinization and re-hydration, the 3-μm paraplast tissue section slides were placed in a plastic jar containing Citrate buffer and then boiled for 20 min by microwave irradiation. Thereafter, sections were incubated with TUNEL reaction mixture in a humidified dark atmosphere for 60 min at 37 °C. Sections were then rinsed and observed.

### Sanger sequencing

CARF mutation was confirmed by Sanger sequencing in the patient and a fertile man with the genomic DNA extracted from peripheral blood. Primers were designed using the Primer 5 program (FW: AGATCGAGGTGCCCTTGTTG & RW: GCCAACTGGGACACACTTGC). Sanger sequencing was performed in General Biosystems (Anhui). Sequencing results were analyzed with Chromas software.

### Statistical analysis

Data statistical analyzes were performed using the two-tailed Student’s *t-*test with GraphPad Prism 8 unless otherwise specified. All result data are presented as means ± SEM, *P* values are presented as follows: *P < 0.05, **P < 0.01, ***P < 0.001.

## Supplementary information

Supplementary Material

## Supplementary information

**Supplementary Information** accompanies the paper at (10.1038/s41421-020-00212-7).
