# Supplementary material for: CARF promotes spermatogonial self-renewal and proliferation through Wnt signaling pathway
Source: Cell Discov. 2020 Nov 17;6:85. doi: 10.1038/s41421-020-00212-7 (PMC7674451; doi:10.1038/s41421-020-00212-7)
Supplement: Supplementary file 1 — Supplementary Material [file 41421_2020_212_MOESM1_ESM.doc]

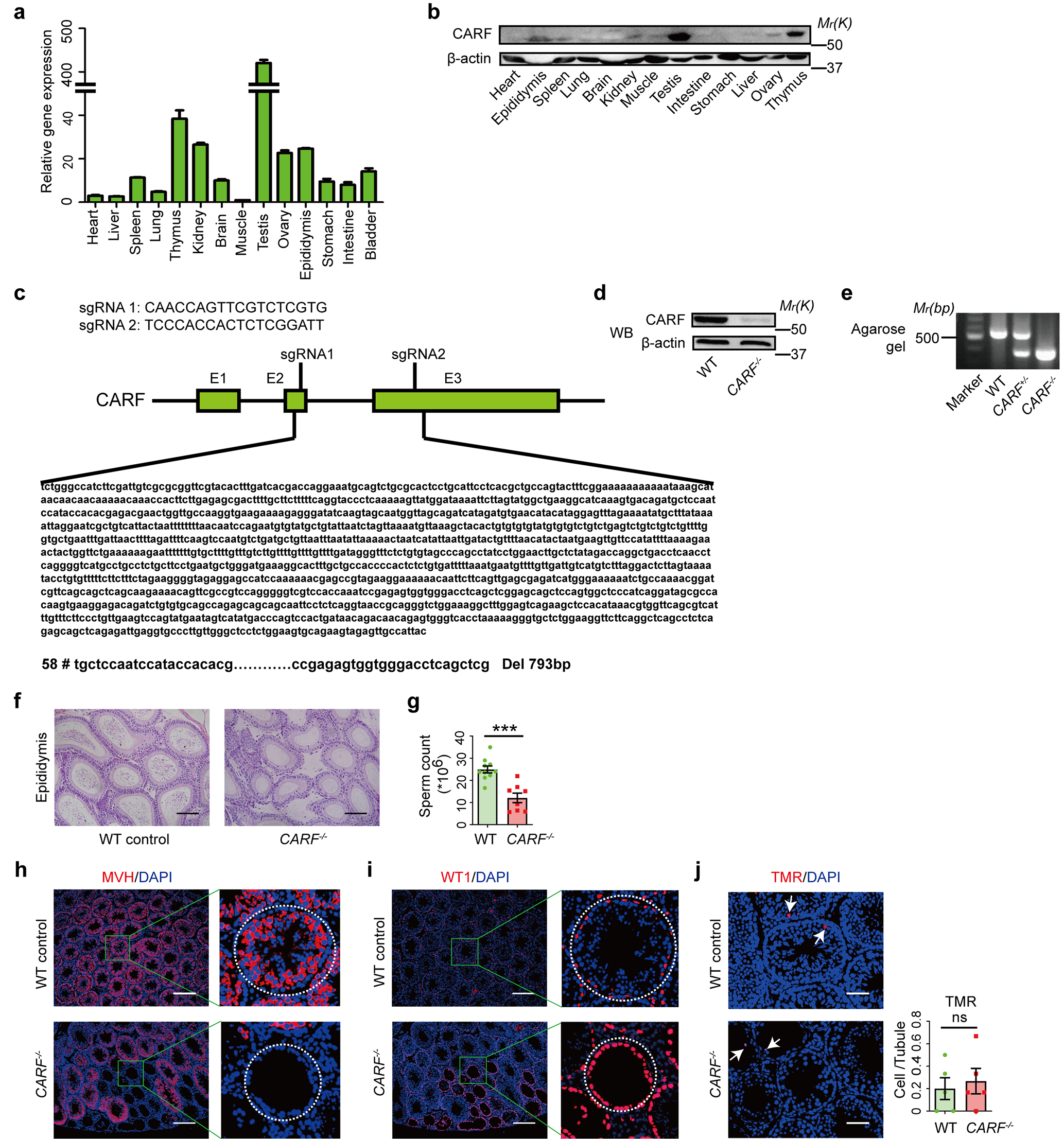


**Figure S1. Expression of CARF among different tissues and gene disruption of the *CARF* locus. Related to Figure 1.**

(a) Quantitative real-time PCR analysis of CARF RNA levels in mouse organs. Data are expressed as mean ±SEM. Fold changes were compared with the heart, and normalized to β-actin.

(b) Western blot analysis of CARF protein levels in WT mouse organs.

(c) Schematic strategy for constructing *CARF* knockout mouse model and the result of sequencing verification. E, exon.

(d) Western blot analysis for CARF from testis of WT control and *CARF^-/-^* mice.

(e) Genotyping of *CARF* knockout mice by PCR.

(f) H&E staining of epididymis sections from WT control (left) and *CARF*^-/-^ mice (right). (Scale bars, 100 μm).

(g) Sperm counts in cauda epididymis from 8-16 weeks old WT control (n=10) and *CARF^-/-^*mice (n=8). Each dot in the graphs represents an individual mouse. Bar graphs represent mean ±SEM. Statistical analysis was performed by two-tailed t test, n.s., not significant,****P*< 0.001.

(h) Immunostaining for germ cell marker MVH (red) in testis sections from 16-week-old WT control (left) and *CARF*^-/-^mice (right), with co-staining for DAPI (blue). (Scale bars, 200 μm). The boxed area is magnified on the right side. The outlines of seminiferous tubules are indicated by dashed lines.

(i) Immunostaining for Sertoli cell marker WT1 (red) in testis sections from 16-week-old WT control (left) and *CARF*^-/-^mice (right), with co-staining for DAPI (blue). (Scale bars, 200 μm). The boxed area is magnified on the right side. The outlines of seminiferous tubules are indicated by dashed lines.

(j) TUNEL staining of testis sections from 16-week-old WT control and *CARF^-/-^* mice (Scale bars, 50 μm). Arrows indicate germ cells in seminiferous tubules that are undergoing apoptosis. Dot graph shows the apoptotic cell numbers per tubule in testis sections from 8-16 weeks old mice (mice, n=5; tubules of one mouse, n≥10). Each dot in the graphs represents an individual mouse. Bar graphs represent mean ±SEM. Statistical analysis was performed by two-tailed t test, n.s., not significant.


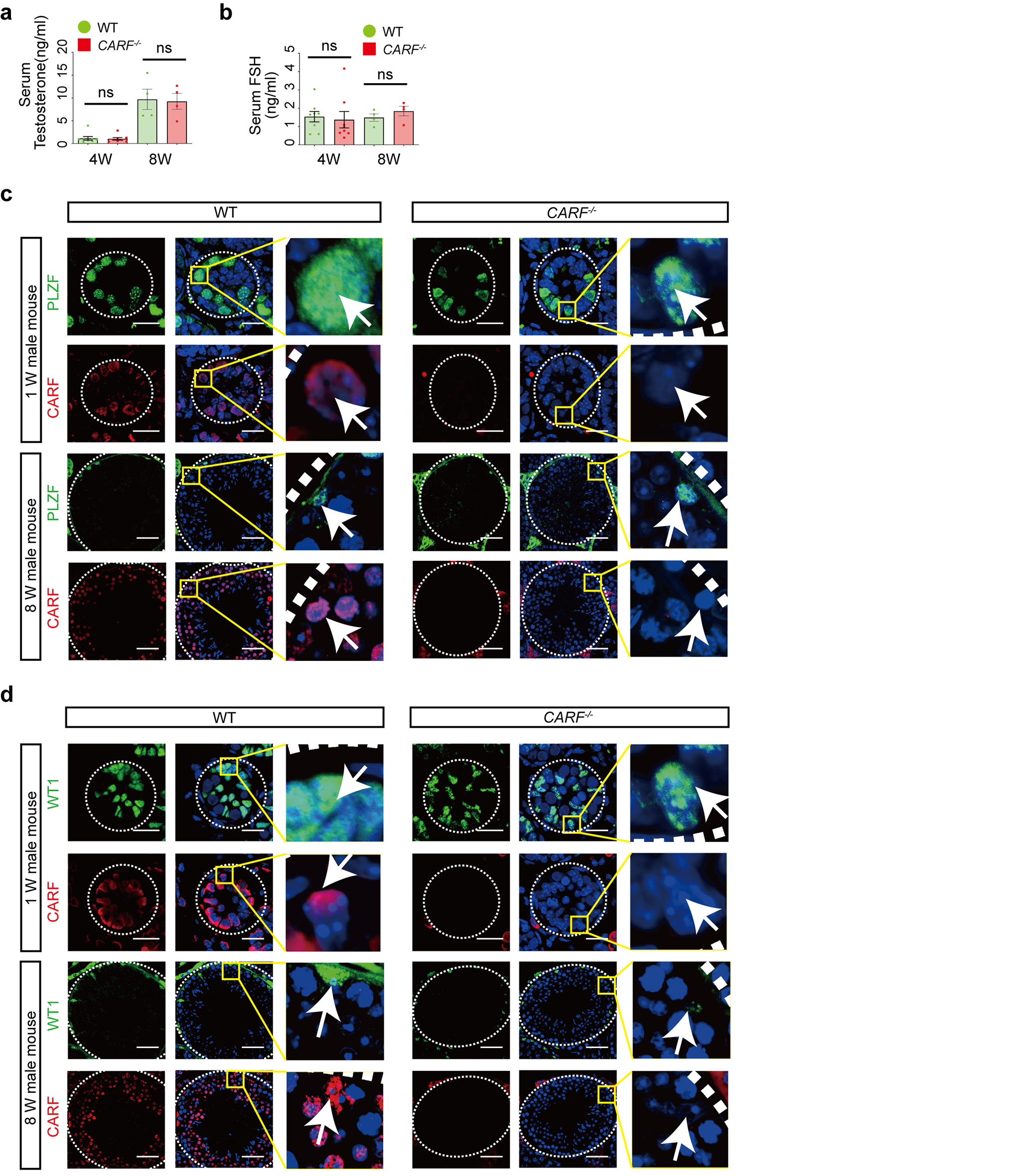


**Figure S2. Hormone levels in WT control/*CARF^-/-^* mice, and CARF expression in Sertoli cells and spermatogonia of WT mice. Related to Figure 3.**

(a) ELISA analysis of serum testosterone concentrations in 4-week-old, 8-week-old WT control and *CARF^-/-^* mice (4-week-old mice, n=8; 8-week-old mice, n=4). Each dot in the graphs represents an individual mouse. Bar graphs represent mean ±SEM. Statistical analysis was performed by two-tailed t test, n.s., not significant.

(b) ELISA analysis of serum follicle-stimulating hormone (FSH) concentrations in 4-week-old, 8-week-old WT control and *CARF^-/-^*mice (4-week-old mice, n=8; 8-week-old mice, n=4). Each dot in the graphs represents an individual mouse. Bar graphs represent mean ±SEM. Statistical analysis was performed by two-tailed t test, n.s., not significant.

(c) Serial cross-sectional images of CARF (red) immunostaining, PLZF immunostaining (green) and DAPI (blue) staining of testis sections from 1-week-old, 8-week-old WT and *CARF*^-/-^ mice (Scale bars for 1-week-old, 20 μm, for 8-week-old, 50 μm). The boxed area is magnified on the right side. The outlines of seminiferous tubules are indicated by dashed lines. Arrows indicate undifferentiated spermatogonia.

(d) Serial cross-sectional images of CARF (red) immunostaining, WT1 immunostaining (green) and DAPI (blue) staining of testis sections from 1-week-old, 8-week-old WT and *CARF*^-/-^ mice (Scale bars for 1-week-old, 20 μm, for 8-week-old, 50 μm). The boxed area is magnified on the right side. The outlines of seminiferous tubules are indicated by dashed lines. Arrows indicate Sertoli cells.


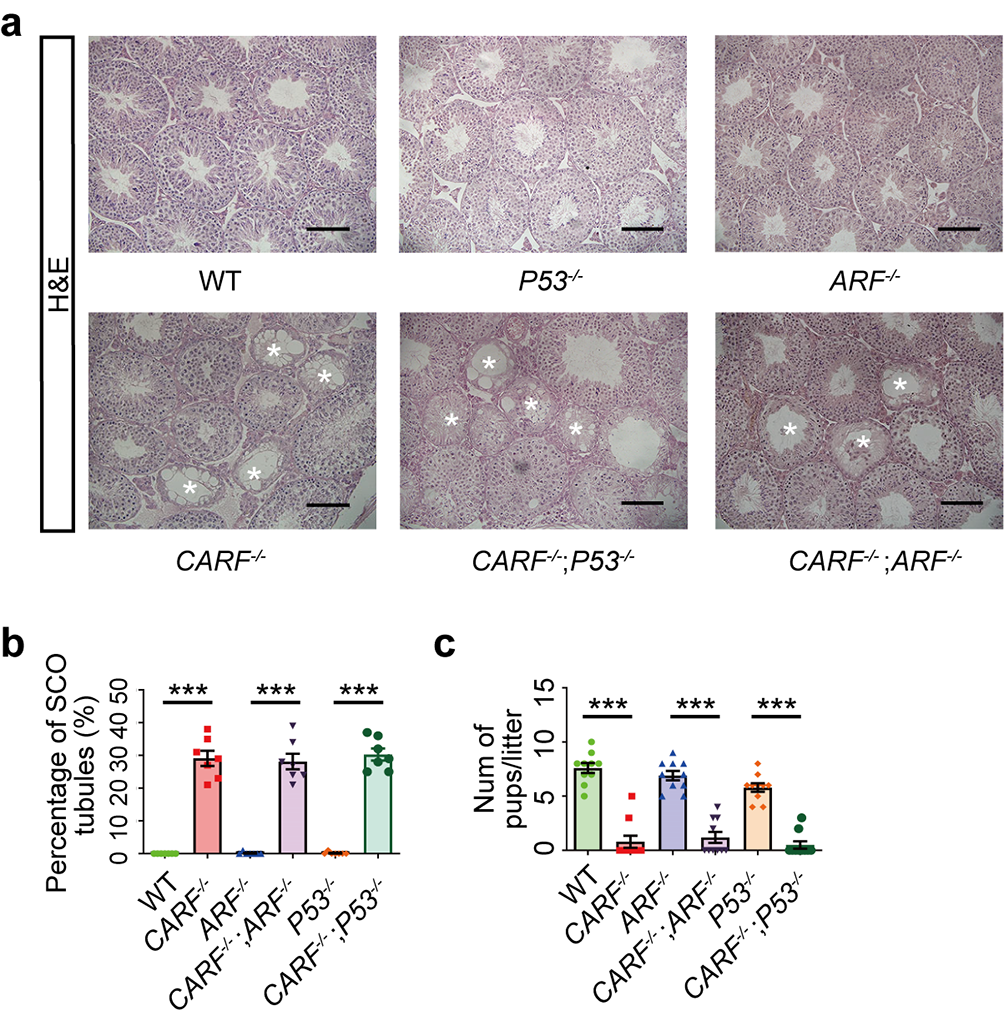


**Figure S3. Reduced fertility in *CARF^-/-^* mice is independent of p53/ARF signaling pathway. Related to Figure 6.**

(a) H&E staining of testis sections from 16-week-old WT, *CARF^-/-^, P53^-/-^*, *CARF^-/-^*;*P53^-/-^*, *ARF^-/-^* and *CARF^-/-^*;*ARF^-/-^*mice (Scale bars, 100 μm). The white asterisk indicates an SCO seminiferous tubule.

(b) Percentage of SCO tubules in testis sections from 8-16 weeks old WT, *CARF^-/-^, P53^-/-^*, *CARF^-/-^*;*P53^-/-^*, *ARF^-/-^* and *CARF^-/-^*;*ARF^-/-^*mice (mice, n=7; sections of one mouse, n>5). Each dot in the graphs represents an individual mouse. Bar graphs represent mean ±SEM. Statistical analysis was performed by two-tailed t test, ****P*< 0.001.

(c) Comparison of male fertility of WT (n=8), *CARF^-/-^* (n=8)*, P53^-/-^* (n=7), *CARF*^-/-^;*P53*^-/-^ (n=7), *ARF^-/^*^-^ (n=10) and *CARF^-/-^*;*ARF^-/^*^-^ mice (n=10). Each dot in the graphs represents an individual litter. Bar graphs represent mean ±SEM. Statistical analysis was performed by two-tailed t test, ****P*< 0.001.


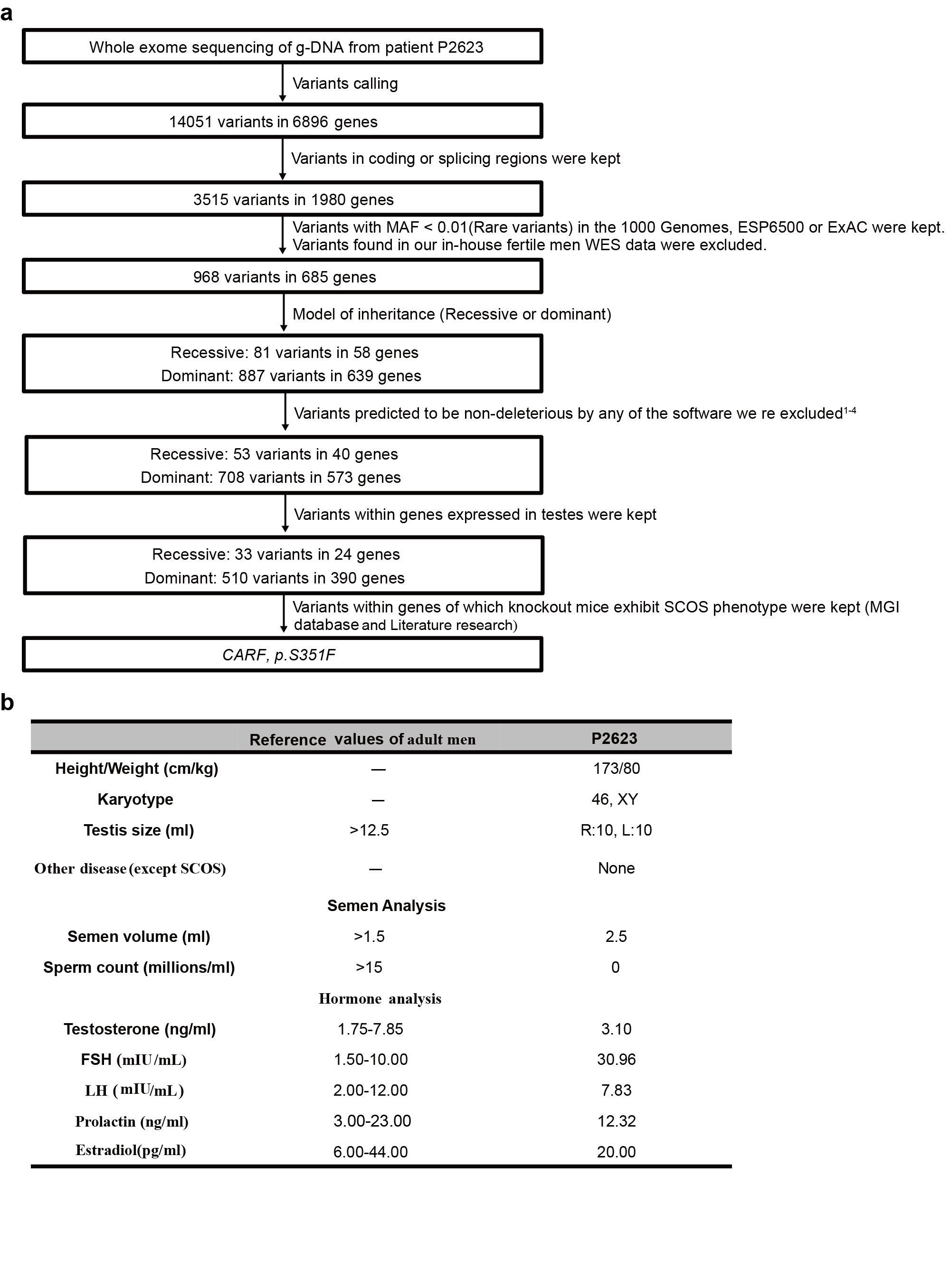


**Figure S4. Clinical sample analysis. Related to Figure 7.**

(a) WES data filtering pipeline for Patient P2623.

(b) Detailed clinical information of P2623 harboring *CARF* p.S351F mutation. FSH, Follicle-Stimulating Hormone; LH, Luteinizing Hormone.

**Supplementary Table S1. PCR primers used in this study, Related to Figure 3, Figure 5 and Figure 7and Supplementary Figure S1.**

Primers for mouse genotyping

| mouse | Forward primer(s) (5'-3') | Reverse primer(s) (5'-3') |
| --- | --- | --- |
| *CARF^+/+^* | GGACTCTTAGTAAAATACCTGTGTTTTTCTTC | GTAATGGCAACTCTACTTCTGCA |
| *CARF^-/-^* | GGACTCTTAGTAAAATACCTGTGTTTTTCTTC | AATCCATACCACACGCCG |

| Gene | Forward primer(s) (5'-3') | Reverse primer(s) (5'-3') |
| --- | --- | --- |
| *m-Actb* | CTGGCTGGCCGGGACCTGACA | ACCGCTCGTTGCCAATAGTGATGA |
| *m-Rps2* | CTGACTCCCGACCTCTGGAAA | GAGCCTGGGTCCTCTGAACA |
| *m-GDNF* | CCAGTGACTCCAATATGCCTG | CTCTGCGACCTTTCCCTCTG |
| *m-Axin2* | TGACTCTCCTTCCAGATCCCA | TGCCCACACTAGGCTGACA |
| *m-CyclinD1* | GCGTACCCTGACACCAATCTC | CTCCTCTTCGCACTTCTGCTC |
| *m-Lef1* | GAGCTTTGTCAAATAAAGTGCC | TGACATCTGACGGGATGTG |
| *m-Wnt6* | CAGTTCCAGTTCCGTTTCC | CAAACACGAAAGCTGTCTC |
| *m-Wnt3a* | TACCCAATTTGGTGGTCCC | ATGTAATTGCGGCAGAAGC |
| *m-Tcf1* | GAGCACACTTCGCAGAGACT | GGCATGAGCAGATTGAAGGC |
| *m-c-myc* | ATGCCCCTCAACGTGAACTTC | CGCAACATAGGATGGAGAGCA |
| *m-Wnt4* | GCAGGAAGGCCATCTTGACA | CGGATGTCCTGCTCACAGAA |
| *m-Lin28a* | TTTGCCTCCGGACTTCTCTG | CGCAGTTGTAGCACCTGTCT |
| *m-Gfra1* | CGGGCAAGGAAACCAACTTC | TTGGAAATGTGTTCCACTGATATG |
| *m-c-fos* | GTGAAGACCGTGTCAGGAGG | GATCTGTCTCCGCTTGGAGT |
| *h-GAPDH* | AGGTCGGAGTCAACGGATTTG | TGTAAACCATGTAGTTGAGGTCA |
| *h-CARF* | GATGAACTGGTTGCCAAGGT | CGAGCTGAGTTCCCACTCTC |
| *h-Axin2* | AGGCTAGCTGAGGTGT | AGGCTTGGATTGGAGAA |
| *h-CCND1* | GCTGGCCATGAACTACCTGGA | TCCATTTGCAGCAGCTCCTC |
| *h-NKD1* | GCTGGCCATGAACTACCTGGA | TCCATTTGCAGCAGCTCCTC |

Primers for RT-qPCR

**REFERENCES**

1 Sim, N. L. *et al*. SIFT web server: predicting effects of amino acid substitutions on proteins. *Nucleic acids research* **40**, W452-W457, (2012).

2 Davydov, E. V. *et al*. Identifying a High Fraction of the Human Genome to be under Selective Constraint Using GERP plus. *Plos Comput Biol* **6**, (2010).

3 Lindblad-Toh, K. *et al*. A high-resolution map of human evolutionary constraint using 29 mammals. *Nature* **478**, 476-482, (2011).

4 Garber, M. *et al*. Identifying novel constrained elements by exploiting biased substitution patterns. *Bioinformatics* **25**, I54-I62, (2009).
